# An integrated system for the management of environmental data to support veterinary epidemiology

**DOI:** 10.3389/fvets.2023.1069979

**Published:** 2023-03-21

**Authors:** Matteo Mazzucato, Giulio Marchetti, Marco Barbujani, Paolo Mulatti, Diletta Fornasiero, Claudia Casarotto, Francesca Scolamacchia, Grazia Manca, Nicola Ferrè

**Affiliations:** Istituto Zooprofilattico Sperimentale delle Venezie, Legnaro, Italy

**Keywords:** GIS, environmental data, Satellite Earth Observation, veterinary epidemiology, support system

## Abstract

Environmental and climatic fluctuations can greatly influence the dynamics of infectious diseases of veterinary concern, or interfere with the implementation of relevant control measures. Including environmental and climatic aspects in epidemiological studies could provide policy makers with new insights to assign resources for measures to prevent or limit the spread of animal diseases, particularly those with zoonotic potential. The ever-increasing number of technologies and tools permits acquiring environmental data from various sources, including ground-based sensors and Satellite Earth Observation (SEO). However, the high heterogeneity of these datasets often requires at least some basic GIS (Geographic Information Systems) and/or coding skills to use them in further analysis. Therefore, the high availability of data does not always correspond to widespread use for research purposes. The development of an integrated data pre-processing system makes it possible to obtain information that could be easily and directly used in subsequent epidemiological analyses, supporting both research activities and the management of disease outbreaks. Indeed, such an approach allows for the reduction of the time spent on searching, downloading, processing and validating environmental data, thereby optimizing available resources and reducing any possible errors directly related to data collection. Although multitudes of free services that allow obtaining SEO data exist nowadays (either raw or pre-processed through a specific coding language), the availability and quality of information can be sub-optimal when dealing with very small scale and local data. In fact, some information sets (e.g., air temperature, rainfall), usually derived from ground-based sensors (e.g., agro-meteo station), are managed, processed and redistributed by agencies operating on a local scale which are often not directly accessible by the most common free SEO services (e.g., Google Earth Engine). The EVE (Environmental data for Veterinary Epidemiology) system has been developed to acquire, pre-process and archive a set of environmental information at various scales, in order to facilitate and speed up access by epidemiologists, researchers and decision-makers, also accounting for the integration of SEO information with locally sensed data.

## 1. Introduction

Changes in climate and human activities, as well as the increasing movements of goods and animals related to globalization of markets, are considered to be among the key factors for the spread of emerging infectious diseases, and of vector borne diseases (VBD) in particular (1). The worldwide expansion of West Nile Virus (WNV) in the last few decades, and its endemisation, perfectly exemplify the variations in the spread of emerging diseases (2). This is also the case of Italy, where WNV reappeared in 2008, after a 10-year hiatus, and has become endemic in an ever-increasing number of regions (3).

Both human and veterinary epidemiology has greatly focussed on studying the environmental factors that contribute to the introduction and spread of animal diseases, especially with zoonotic potential (4–6). In particular, the need of understanding and accounting for local environmental features is acquiring relevance for epidemiologists, decision makers, and health operators, to both improve and fine-tune specific monitoring activities and surveillance plans (7–11).

The main challenges when working with environmental data are the collection, organization, and analyses of satellite data. In fact, difficulties in accessing and using data can occur due to potential differences between what satellite sensors actually measure and what information is of interest either for decision-making or for inclusion in ecological and epidemiological analyses (12). Other problems that could arise include technical issues, such as the interference of clouds, and lack of technical and cross-funding resources (13, 14). In addition, there are further barriers related to the perception of 'readiness for use' and permissible levels of uncertainty among different types of users, such as those with the technical capabilities to process remotely sensed data and end-users of indicators obtained from satellite data (15). The increasing availability of environmental datasets does not always translate into a widespread use for scientific research. Although most of the data-users belong to the public sector, and Universities in particular, the number of downloads from this category is very limited (16), suggesting that most users tend to register to acquire few data. These project-oriented downloads indirectly imply spending a significant amount of resources in the data gathering process (i.e., searching, understanding and filtering data).

Although the phase of data acquiring has become easier than in the past also thanks to specific web portals (e.g., United States Geological Survey's *Earth Explorer*; European Union's *Copernicus Open Access Hub*; European Union's CMEMS), most of them only allow a few or individual downloads at once. Hence, multiple downloads require huge “manual work”, or some coding skills to automate the process with scripts, whose implementation may be time-consuming. Furthermore, epidemiological researches tend to rely on a bundle of commonly used environmental variables (such as temperature, precipitation, plant biomass, humidity, etc.), whose extraction requires at least some basic GIS skills. In this context, we present an instrument that might help to fill the gap between researchers and the most common environmental datasets.

Nowadays, there are online services that offer the possibility of obtaining ready-to-use satellite data; one of the most widely used is Google Earth Engine (GEE) (17), which, through a programming language, can give access to a vast catalog of data, obtained from different sources, that can be further processed directly in the cloud. Although they are very powerful, they may have certain limitations, which may not be acceptable to a public administration (18).

Given these limitations, the *Environmental data for Veterinary Epidemiology* system (EVE) has been recently developed and implemented at the *Istituto Zooprofilattico Sperimentale delle Venezie* (IZSVe), an Italian public health institute that conducts control and research activities in the fields of animal health, food safety and zoonoses. EVE acquires, processes and stores a set of environmental information that can be directly used for the evaluation of epidemiological aspects in the veterinary field. Although EVE provides some of the functionalities already present on other accessible online platforms, it is mainly tailored to feed epidemiological analyses with data obtained *via* agreements with local agencies and with a much higher geographical scale than most of the freely available satellite imagery. Furthermore, EVE includes a series of procedures to enhance the temporal resolution and reconstruct data that might be missing in unprocessed environmental layers.

Hereby we illustrate EVE and its main components, and the characteristics that make the system particularly useful in studies aimed at integrating satellite derived information and data obtained from ground sensors on a very small scale. Although EVE is not the only available applications, it can be a starting point for similar tools, when there are comparable limitations and needs. This study is not intended to publicize a commercial product, but rather as a sort of guidelines for anyone who needs to implement a similar system and could benefit from our experience.

## 2. Materials and methods

### 2.1. Geographical levels

**Figure 1:**
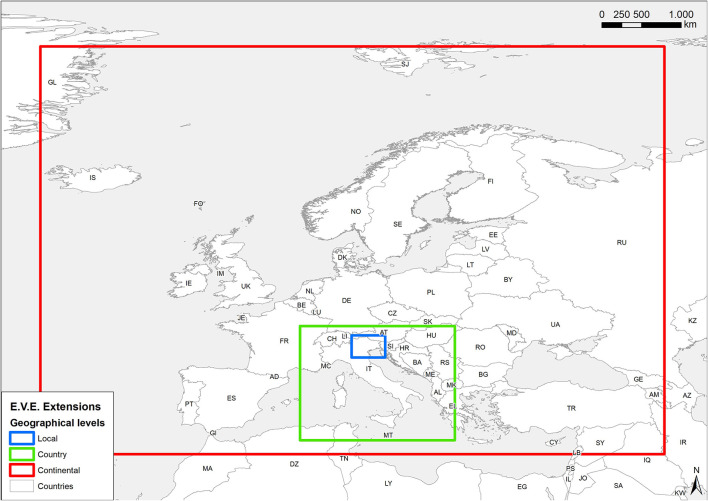
Extent for different geographical levels: local, country, continental.

The data elaborations and storage have been designed according to three different geographical levels (Figure 1) which varied for extension, spatial and temporal resolution, and Coordinate Reference System (CRS).

A) Local level, centered on the north-eastern Italian regions (i.e., Veneto, Friuli Venezia Giulia and the autonomous provinces of Trento and Bolzano), hereafter also “Triveneto”; the level contains remote sensed data at the highest spatial and temporal definition, and local ground sensed data (i.e., meteorological stations), in EPSG:32632 - UTM32N (hereafter UTM32N) CRS;
B) Country level (i.e., Italy); contains remote sensed data with a lower resolution in UTM32N CRS;
C) Continental level (i.e., western Europe); contains a limited set of remote sensed data with the lowest resolution, in EPSG: 4326 - WGS84 (hereafter, WGS84) CRS.

Levels A and B contain data derived from different providers, the grids were resampled to spatial resolutions that are multiples or submultiples of 1 km, and aligned to a unique main grid, choosing the resolution value closest to the resolution of the input data. Conversely, level C uses another CRS and does not spatially overlap with the previous levels.

The choice of the different scales derives essentially from the experiences gained from previous projects that have included environmental data and from the specific requests of the Epidemiologists/researchers. Triveneto (local level) is the area of specific competence for IZSVe and Italy is defined in accordance with the Country level for research which involves other IZS or reference laboratories. The continental level covers most of the European Countries and it has been previously included in works with neighboring Countries.

Relevant literature was investigated to define the potential set of climatic and environmental drivers of diseases/species, accordingly to the needs of epidemiologists/researchers at the IZSVe (i.e., for Avian Influenza, and WNV). The main environmental data catalogs have been queried for both gridded datasets and point data from ground sensors, to assess their availability and completeness. International catalogs ranging over continental (e.g., Copernicus Open Access Hub) or global scale (e.g., Earth Explorer) have been considered as main sources, as well as different web portals or services of the Regional Agency for Environmental Protection (*Agenzia Regionale per la Prevenzione e Protezione Ambientale*- ARPA) have been used as local level. Not all data were available as “open access” and some required a previous registration or, in some cases, a written request to data owners.

The acquisition of each type of data has been implemented as specific, automated procedures directly from web repositories. The downloading process has been followed by specific operations of data harmonization. Workflows for different environmental data or data sources are different, but as an overall principle we have chosen a unique precise output structure.

**Figure 2:**
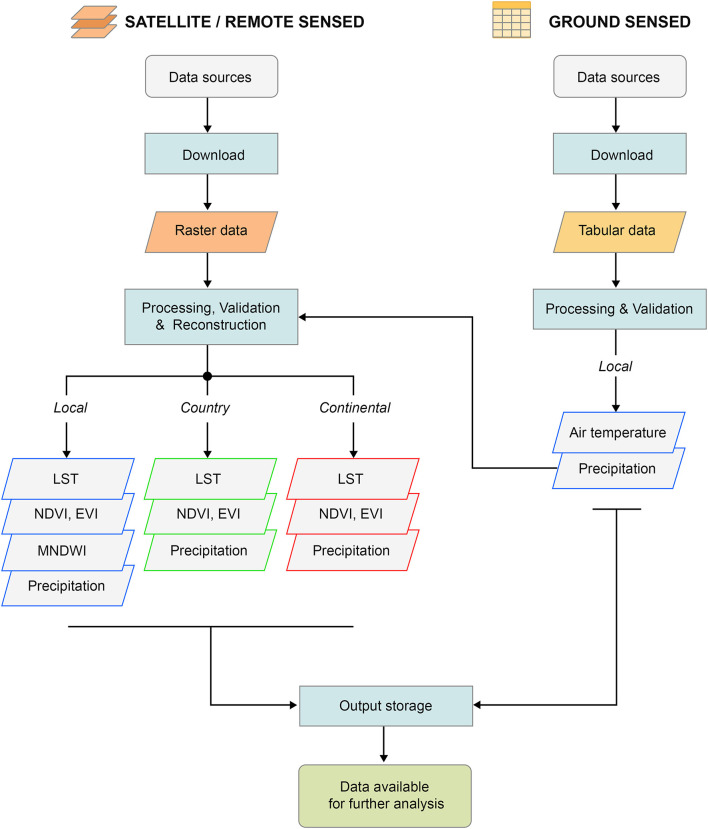
General framework representing the flow at macro-level operated by the EVE system to produce the final dataset.

Figure 2 reports the general workflow for both Satellite and Ground sensed data.

### 2.2. Acquisition and processing of the satellite data

**Figure 3:**
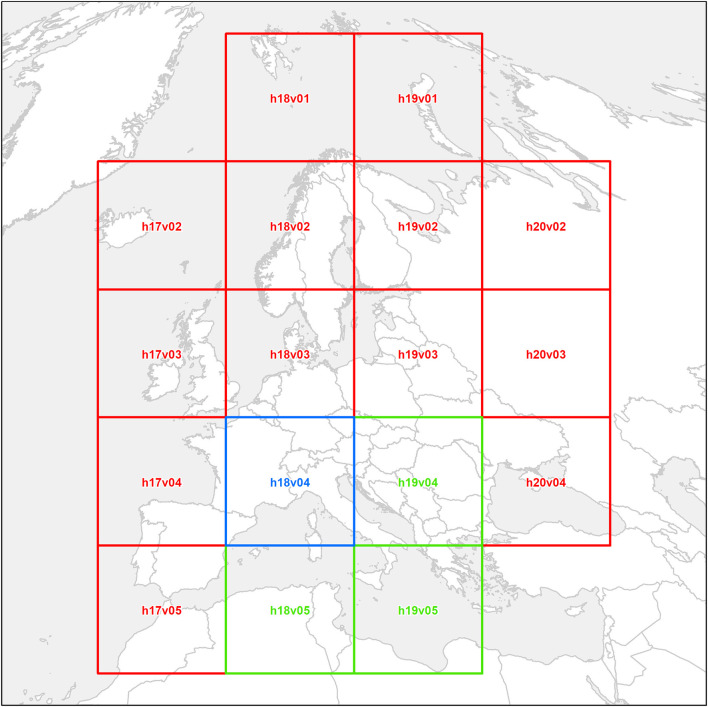
Terra and Aqua satellites footprints selected for the Local (blue), Country (blue and green), and Continental (blue, green and red) levels.

Land Surface Temperature (LST) is derived from Earth emissivity, therefore it is available from multiple sources (19–21). LST is considered being correlated with air temperature, which has been demonstrated being an environmental driver of WN fever outbreaks (22, 23). EVE uses satellite products from the NASA *MODerate resolution Imaging Spectroradiometer* (MODIS) on board the satellites TERRA (20) and AQUA (24) (Figure 3). MODIS LST data have a temporal resolution up to four images per day, included in the MOD11A1 and MYD11A1 products (named M^*^D11A1 hereafter), and other aggregate products are also available, as the 8-days averaged LST (M^*^D11A2). In EVE, LST datasets for both Continental and Country levels derive from M^*^D11A2 products, which are extracted, mosaicked and filtered with the Quality Control band (QC) in order to produce good quality raster images with a spatial resolution of 1 km and temporal resolution of 8 days. On the other hand, the dataset for the Local Level is modeled and reconstructed from original MODIS daily data (*i.e.*, M^*^D11A1), as illustrated by Neteler (25). In particular, LST data are cleaned through the QC band, and filtered to eliminate outliers accounting for: (i) pixel values distribution and (ii) typical altitudinal LST gradients (i.e., the inverse relationship between temperature and altitude). Filtered data are then modeled and reconstructed on the base of the 200 m Digital Elevation Model (DEM), to produce a higher-resolution, gap-free time series of four images per day, aligned with the other UTM32N gridded data.

**Figure 4:**
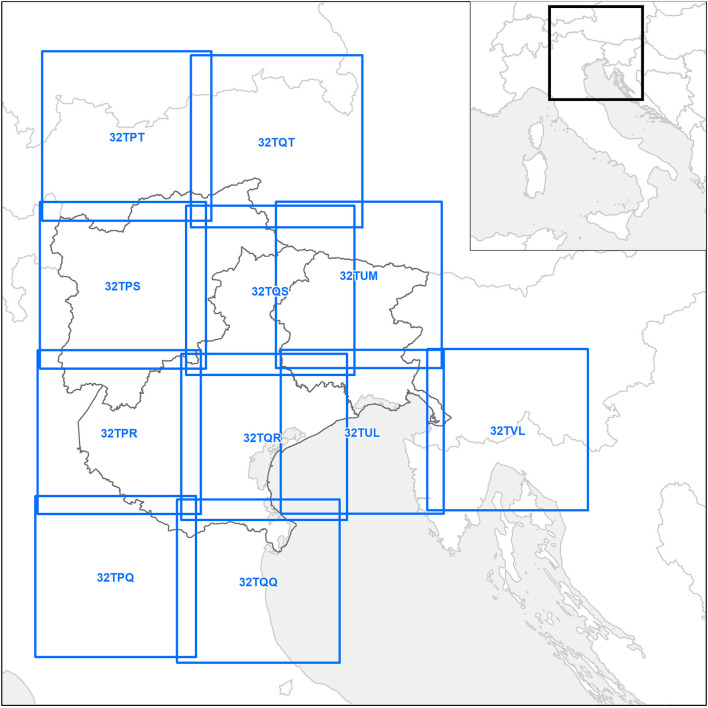
The Sentinel-2 satellite footprints selected for the Local level (blue).

The Normalized Difference Vegetation Index (NDVI) is an index of the phenological state of vegetation and has been used in relation to several infectious and vector borne diseases [e.g., rabies and malaria (26, 27)]. However, the reliability of NDVI could be hampered due to canopy background (e.g., in densely forested areas), soil type and specific atmospheric conditions (28, 29). The Enhanced Vegetation Index (EVI) can be used to tackle these issues (30), with the drawback of being potentially affected by topographic effects (31). The NDVI and the EVI data over Continental and Country levels derive from the MODIS MOD13A2 product, in which the vegetation indexes are contained as two separate bands (32). The original spatial resolution is 1 km imagery, with a temporal resolution of 16 days. In this dataset, each pixel has the maximum value of vegetation index recorded in the period of reference, in order to minimize the risk of capturing clouds. Similarly to LST, both vegetation indexes are extracted from the original Hierarchical Data Format (HDF) files, mosaicked and filtered with the QC band. Furthermore, averaged images are computed from two consecutive images in order to temporally align the dataset to 8-days LST data (M^*^D11A2), thus obtaining raster images with a spatial resolution of 1 km and temporal resolution of 8 days. Conversely, the vegetation indices for the Local level are derived from the high-resolution imagery of Copernicus's Sentinel-2 satellites, available starting from 2015, with an original spatial resolution of 10 m (33). The Sentinel-2 constellation, composed of two satellites, completely covers the Local level area every 5 days, with two intermediate passages on the eastern and western half of the study area (Figure 4). Only images with a cloud-cover lower than 80% are considered as eligible for the download and, once downloaded, the original bands in JP2 format are filtered, transformed and mosaicked over the study area. Finally, the vegetation indexes are computed from the Red, Near Infrared, and Blue bands (the latter for EVI only). Clouds and cloud shadow pixels are removed according to the Scene Classification Layer (SCL) raster, downloaded with the tiles. The final raster images are rescaled to a resolution of 200 m, thus being aligned with the other UTM32N data.

The Modified Normalized Difference Water Index (MNDWI) is aimed at detecting water bodies (34), providing indication on potential breeding foci for arthropod vectors. It is computed for the Local level only, being obtained from Sentinel-2 imagery with a process similar to the NDVI and EVI layers for the Local level, and it is derived from the Green and Shortwave Infrared bands.

Precipitation is a weather data that can influence the abundance and distribution of arthropod vectors (35). The datasets (Country and Continental levels) derive from both the E-OBS dataset, provided by the European Climate Assessment and Dataset project (for the 2010-2014 period), and the NASA Global Precipitation Measurement mission (GPM; for years since 2015). E-OBS data is a gridded dataset obtained from interpolation of precipitation recorded by a network of European meteorological stations (36). The version 16.0 of E-OBS has a daily temporal resolution and a spatial resolution of 0.25 degrees (about 27 km). The GPM dataset derives from satellite-based estimation of precipitations (37). The selected product is the daily GPM_IMERGDF_05 dataset, with a spatial resolution of 0.1 degrees (about 10 km). EOBS and GPM data are extracted from NetCDF files, transformed into raster image and rescaled to the nearest multiple of 1 km (27 km for E-OBS-derived images and 10 km for GPM). These variables and indices represent the baseline environmental and climatic drivers that can significantly influence the ecology and dynamics of arthropod vectors or pathogens (38), as well as their potential interactions (39). Therefore, the environmental and meteorological conditions act as important drivers of spatial and seasonal patterns of infections, also affecting epidemic emergences, and shifts in the at-risk populations. Changes in climate conditions (e.g., global warming, increased flooding and drought events, desertification) can lead to a marked loss in biodiversity and to more suitable conditions for the proliferation of arthropod vectors, but also for food and water-borne diseases (5, 40). A summary of the products obtain by the dataset are present in Table 1.

**Table 1:** Summary of the EVE dataset as of August 2022.

**Product**	**Geographical levels**	**Source**	**Spatial resolution**	**Temporal resolution**	**N. Images**	**Min date**	**Max date**
LST	Local	AQUA/TERRA (MODIS)	200 m	Daily	18,140	01-01-2010	27-07-2022
LST	Country	AQUA/TERRA (MODIS)	1 km	8 days	2,304	01-01-2010	12-07-2022
LST	Continental	AQUA/TERRA (MODIS)	1 km	8 days	2,302	01-01-2010	12-07-2022
NDVI	Local	SENTINEL-2	100 and 200 m	<5 days	1,094	04-07-2015	22-07-2022
NDVI	Country	TERRA (MODIS)	1 km	9 days	552	01-01-2010	26-06-2022
NDVI	Continental	TERRA (MODIS)	1 km	8 days	552	01-01-2010	26-06-2022
EVI	Local	SENTINEL-2	100 and 200 m	<5 days	1,057	31-12-2015	22-07-2022
EVI	Country	TERRA (MODIS)	1 km	8 days	552	01-01-2010	26-06-2022
EVI	Continental	TERRA (MODIS)	1 km	8 days	552	01-01-2010	26-06-2022
MNDWI	Local	SENTINEL-2	100 and 200 m	<5 days	1,093	04-07-2015	22-07-2022
Precipitation	Local	Meteo stations interpolation	1 km	Daily	4,383	01-01-2010	31-12-2021
Precipitation	Country	E-OBS	27 km	Daily	1,826	01-01-2010	31-12-2014
Precipitation	Continental	E-OBS	27 km	Daily	1,827	01-01-2010	31-12-2014
Precipitation	Country	GPM	10 km	Daily	2,373	01-01-2015	30-06-2021
Precipitation	Continental	GPM	10 km	Daily	2,373	01-01-2015	30-06-2021
DEM	Local	Copernicus	25m, 100m		1		
Land Cover	Continental	CORINE 2006, 2012, 2018 (versions: 2020_20u1)	100 m		3	01-01-2006	01-01-2018

### 2.3. Acquisition and processing of ground sensed data

Precipitation data (Local level) are obtained from spatial interpolation of daily data from meteorological stations, available from four public institutions: the two ARPA of Veneto (ARPAV) and of Friuli Venezia Giulia (ARPAFVG), plus the two Autonomous Provinces of Trento (TN) and Bolzano (BZ). All downloaded data are harmonized, joined to their respective georeferenced station, and interpolated. The output resolution of daily images is set to 1 km, thus being temporally and spatially aligned with the other UTM32N datasets.

The time series of ground sensed data for the local level area are stored in EVE in table format. The core datasets include: (i) daily maximum, average and minimum air temperature at 2 m above the sea level (asl) (named TMX2, TMD2 and TMN2 respectively); (ii) daily precipitation (PREC); and (iii) maximum, average and minimum air relative humidity at 2 m asl (UMX2, UMD2 and UMN2). Meteorological data are acquired separately from their respective regional/provincial services and harmonized in order to obtain a table with a common structure. Harmonized data are cleared from possible errors and are loaded in the EVE database, combined with information regarding Region, acquisition date and codified meteorological variables. Tables can be queried to obtain information for each ground sensed station and used for further elaboration (e.g., the precipitation values are required to interpolate values through the GRASS GIS “spline” algorithm to obtain precipitation over a continuous surface). A summary of the products obtain by the dataset are present in Table 2.

**Table 2:** List of information acquired by ground station as of August 2022.

**Product**	**Geographical levels**	**Source**	**Temporal resolution**	**N. Records**	**Min date**	**Max date**
Air temperature	Local	ARPAFVG, ARPAV, Meteo Trentino, Meteo Alto Adige	Daily	3729835	01-01-2008	31-12-2021
Precipitation	Local	ARPAFVG, ARPAV, Meteo Trentino, Meteo Alto Adige	Daily	1483680	01-01-2008	31-12-2021

### 2.4. Other products

Other geographical data are also stored and available in the EVE, even though they are not processed by the system. The 2011 version of the DEM from the satellite constellation named ‘Satellite Pour l'Observation de la Terre' (SPOT), with the original spatial resolution of 25 m, was resampled and used in the processing of the environmental data (e.g., DEM values are used in the linear model to define LST outliers boundaries and reconstruction of missing data).

Different versions of Corine Land Cover (CLC) (https://land.copernicus.eu/pan-european/corine-land-cover) are available as well in the system and a set of procedures have been implemented to calculate the percentage of land cover in specific areas.

Furthermore, other information was derived from EVE's datasets: the variability of LST/vegetation indices/MNDWI values over an interval of time (with Standard Deviation and Kurtosis); the number of rainy days, or days exceeding a certain threshold of precipitation; the Growing Degree Days (GDD), calculated from temperature records (41); and the De Martonne Index of aridity (DMI), combining precipitation and temperature (42) to estimate potential drought conditions that can influence the habitat of larval and adult mosquitoes (43).

Using the daily LST and Precipitation data covering the Local level, we have produced raster images representing the monthly average values for both precipitation and temperature, which were then used to create a yearly image of the 19 bioclimatic variables defined by the “biovars” function for the years between 2010 and 2020, using the R library “dismo” (44).

### 2.5. System implementation

EVE has been implemented with a modular approach, and is the result of several sequential steps, ranging from data gathering, processing, storing and querying. The starting point is represented by the different download and output production workflows, while the querying procedures have been defined later. All the output gridded datasets and their assets are archived on a redundant file server, while most of the raw input data are not saved because of their elevated volume. On the other hand, the data from meteo stations are saved on a dedicated schema on an Oracle^®^ 11g Database Management System (https://www.oracle.com/).

All the main automated procedures have been implemented as scripts in the R software 3.3.5 (45) core with some packages to manage: spatial data [raster (46), rgdal (47), ncdf4 (48)], interaction with database [rjdbc (49)] and management download and parsing of the data from the web [httr (50), XML (51)]. Some of the scripts involving MODIS raw data extraction and reprojection (for LST, EVI and NDVI workflows) derive from the original code kindly made available by Hengl (52), with specific adaptation to our study areas. EVE's R procedures also involve third party softwares and their extensions: GRASS GIS (53), used for LST and precipitation modeling at Local level, particularly the *v.surf.rst* tool (https://grass.osgeo.org/grass82/manuals/v.surf.rst.html); the Modis Reprojection Tool (MRT), specific for MODIS data extraction and transformation from original HDF files (54). Moreover, the download and pre-processing of Sentinel-2 imagery is done using the library “sen2r” of the R software to automate the entire process of image acquisition and processing (55). Finally, an internal catalog has been implemented as a web application developed in HTML5, PHP5.6, and JavaScript, allowing users to easily search and filter data.

## 3. Results

**Figure 5:**
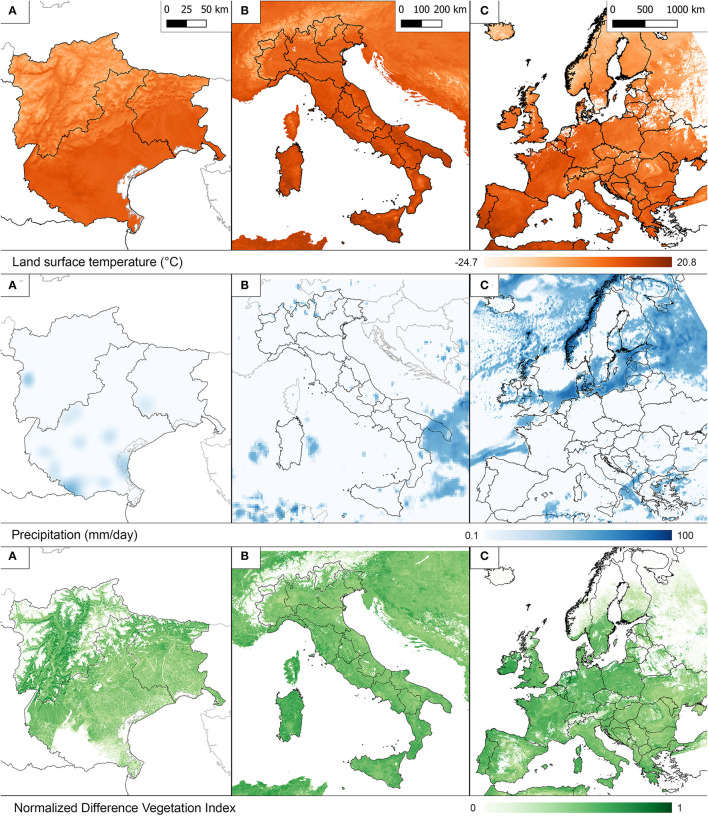
Examples of Land Surface Temperature, Precipitation and NDVI at the three geographical levels: **(A)** Local, **(B)** Country, **(C)** Continental. Displayed data referred to MOD11A1 (day) on 12 January 2020 for local level, MOD11A2 (day) on 09 January 2020 for country and continental levels. To better represent Precipitation at all geographical levels a base-10 logarithmic scale was chosen. In the NDVI legend values were bound to 0–1 range (and not the complete −1–1 scale) to facilitate visualizing the vegetation changes.

As of August, 2022, all EVE's gridded datasets cover the whole 2010–2021 period (an example of the LST raster is reported in Figure 5). There are 40,984 ready-to-use raster images, with a total volume around 650 Gb (859.3 Gb if assets and some of the raw data are also included). Table 1 shows the details of the gridded datasets. The number and type of products are consistent on the three different scales, with the exception of MNDWI. LST are in Celsius degrees, while precipitation data are expressed in millimeters of cumulative rainfall per day.

**Figure 6:**
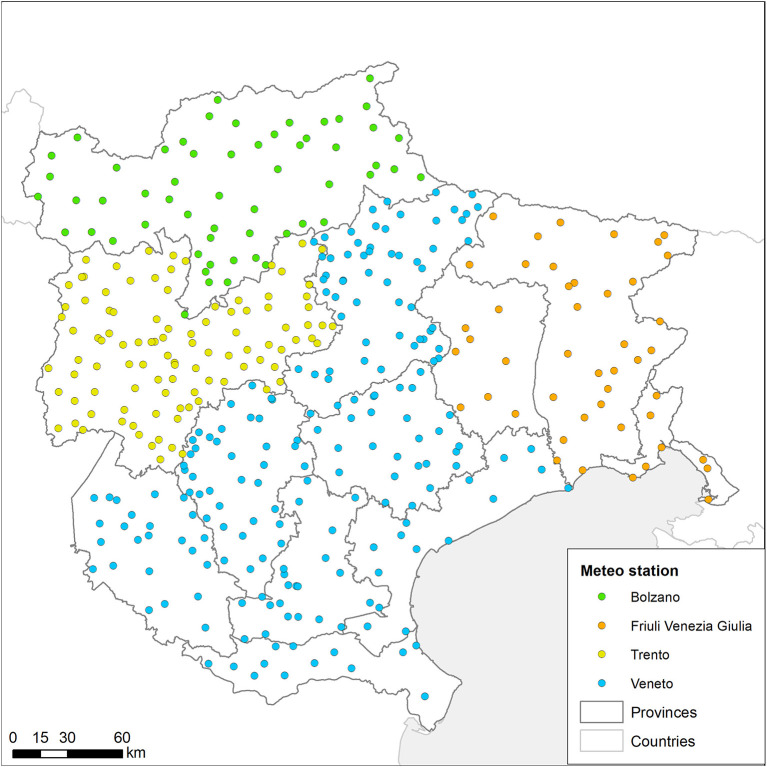
Ground sensed data derived from meteo stations located in different regions and autonomous provinces.

A total of 413 meteorological stations (Figure 6), 398 of which were active in 2021 (199 in Veneto, 48 in FVG and 166 between TN and BZ) are included in the EVE dataset, consisting of almost eight million records stored in the dedicated Database schema. Table 2 shows the details of the gridded datasets. The number and type of products vary among regions, with most stations provided with sensors that record air temperature (maximum, minimum and average values in Celsius degree) and precipitation (millimeters of cumulative rainfall per day). Other products, such as air humidity, are seldom available outside the Veneto Region.

If required, specific indexes (GDD, De Martonne aridity index or biovars) are produced from the dataset already produced by EVE. If necessary, those new outputs are added to the system automatization.

**Figure 7:**
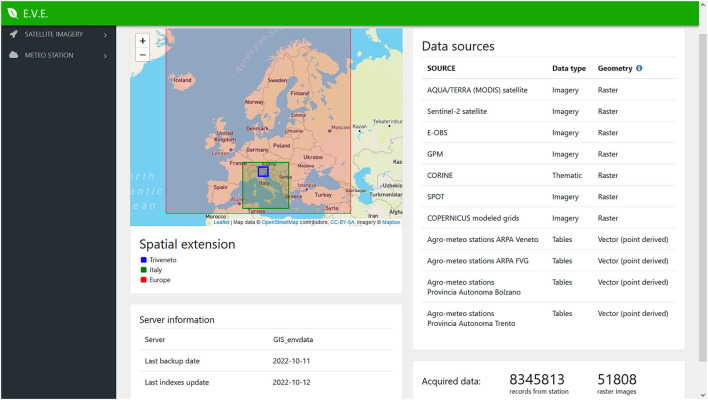
Web application used as catalog of EVE data. It presents both satellite images and ground sensed dataset.

The EVE web application (Figure 7) allows a quick search and filtering of gridded data, based on an updated list of all the available imagery; it also provides information about the meteorological stations included in the system, such as the sensors active for a specific period. At the moment, the web application is available for IZSVe internal users only, and it is used as a data-catalog listing the available datasets that, in combination with the data-control pipeline (not described in this paper), allows to assess the completeness of the information in EVE.

No data policy is currently implemented, given that these data are for internal use only and/or for partners in specific projects.

## 4. Discussion

The EVE system has been developed over a period of about 12 months, during which the developing team was also involved in a series of other projects and tasks, and became fully operative in 24 months. Although the system was initially designed mainly to support the epidemiological analyses of VBD, EVE was developed to provide data to feed any type of eco-epidemiological analyses in the remit of the mission of IZSVe. Therefore, the complexity resulting from the broad scope of the EVE system utilization prompted the project team to design an optimized solution that could accommodate a wide array of preferences in accuracy, level of aggregation, spatial and temporal resolution, and comparability across different regions and data layers. Human public health threats, such as the COVID-19 pandemic and other diseases of potential zoonotic origin, highlighted the key role of veterinary epidemiology and its tools for the development of prevention strategies according to a One Health approach. The One Health concept (56), has become even more important nowadays as many factors have strengthened the interactions between animals, humankind and the environment. For example, the expansion of the human population and its activities into new geographic areas along with climate and land use changes, such as deforestation, intensive farming practices and unsustainable agriculture, are leading to disruptions of natural habitats (57, 58). As a result of these changes, diseases can rapidly spread or evolve unexpectedly, also through spillover of pathogens from animals to humans (59). Consequently, the evolution of the transmission dynamics of such diseases can become complex to investigate. While the availability of data obtained through technologies such as digital maps and remotely-sensed imagery allows exploring the temporal and spatial patterns of the diseases, the wide array of available information poses great challenges for data collection, organization and analysis (60). In this framework, the development of a system for promptly providing environmental and climatic data has become an essential tool to perform complex epidemiological analysis on the interaction between pathogens, host populations, and environment. As a result, EVE consists of a rich bundle of ready-to-use datasets, and it has already proven to be a suitable and convenient tool for saving time during the phases of extraction and preparation of the environmental data needed for further epidemiological and ecological analysis (61–63). The system provides data in multiple formats to its users (e.g., table, raster imagery), so that researchers can easily acquire the pre-processed data in the most convenient format that best suits the type of study they are conducting, without the need of learning complex raw data processing mechanisms, that would otherwise be required prior to analyses.

Although the data availability and computational power do not reach the same levels of some more modern cloud-based platforms, such as GEE, the EVE system presents some unique features, that make its use more convenient for specific users:

- No limit in the size of the downloaded data, allowing the acquisition of all the data needed for the analysis, also through automatic frameworks;
- The use of internal databases, which have data already structured according to the needs of end-users, also complying with internal institutional policies on security levels;
- A wider temporal coverage for the Sentinel-2 catalog imagery compared to GEE (first data available on GEE: 28 March 2017 vs. first Sentinel-2 images on EVE: 4 July 2015).

Moreover, the data included in EVE are harmonized for both temporal and spatial resolutions within each geographical level, permitting to query and compare several variables referred to the same location. All these operations have been standardized, and can be rapidly performed exploiting the naming convention, which allows users to search for images by variable type, extent, date and (in the case of LST) hour. However, the spatial resolution of the outputs is proportional to the scale of work (the larger the scale, the finer the details). Therefore, researchers are restricted to an aprioristic dataset, as it is not recommended to mix data at different scales, without first further processing. In fact, for some of the variables different data sources are used to produce final dataset at various geographic levels (e.g., rainfall on a local scale is obtained from local meteo-stations, while on a continental scale from GPM mission), hence, possible inconsistencies night arise when different geographic levels are compared.

Systems such as EVE have to be constantly updated. This entails not only the need to rely on the “temporal” continuity of the sensors (i.e., the same types of data can be constantly retrieved from the same sensor), but also the need to justify the employment of resources for updating and maintaining the system, by including it or its products in research activities. Therefore, once deemed fully functional, the system was tested in two scientific studies, achieving excellent results. EVE was first exploited in an epidemiological study on Avian Influenza (AI), within the project “Dynamics of avian influenza in a changing world” (EU Horizon 2020 Grant Agreement no. 727922). The second research involving EVE datasets dealt with ecological aspects of West Nile Virus (WNV). In particular, the study aimed at investigating the fluctuations of *Culex* spp. mosquitoes population dynamics in North-Eastern Italy between 2010 and 2018 in relation to environmental variables already available in EVE or calculated *ad hoc* (61). Since then, the system has regularly provided data to analyse and characterize the epidemiological evolution of diseases and viruses such as WNV and AI (61, 62), as well as to provide support for defining risk-based disease control activities.

The above-mentioned case studies are only examples of the large number of applications of the EVE system in terms of methodologies and data analyses. Environmental data, indeed, could be exploited in several types of studies. For example, spatial epidemiology studies the variation of risk factors of diseases in space, allowing to investigate the relationship between environmental factors and the disease incidence in a population (64). It can also be used for projections of the remote-sensed variables that are most strongly associated with a vector/reservoir/disease distribution, to other geographical areas or future times, to forecast disease risk and guide the application of control measures and interventions. Ecological niche modeling can be used to identify environmental factors that shape the spatial distributions of species, to predict the invasive potential and the effects of climate change and land use on species distributions (65). Other examples of how environmental data can be exploited include disease ecology and biogeography, which focus on pathogens distribution and abundance across different scales. The former refers to disease dynamics at a local level in limited time periods, while biogeography expands the study over larger geographical areas and prolonged time windows (66). Beside the wide availability of analytical methods, there are many diseases whose ecology study demands the use of environmental data, and can therefore be investigated with the support of systems such as EVE. A few examples are the already mentioned zoonotic VBDs (e.g., Malaria, WNV, Usutu, Eastern equine encephalitis, and Lyme disease), whose ecology and emergence are deeply intertwined with climatic conditions (temperature, precipitations, aridity, ecc.) and environmental (NDVI, EVI, land use, water availability, ecc.), but also non-zoonotic diseases (bluetongue, African swine fever) that are becoming increasingly important as a consequence of climate changes, and movements of livestock and wild populations. The analysis aimed at studying the influence of environmental and anthropogenic variables on the risk of AI spread among poultry farms, in study area located in Italy (62).

The EVE system offers a set of functionalities for the management of environmental data to be used by researchers as the starting point for analyses, providing data in pre-defined formats through a semi-automatic procedure, allowing investigators to avoid wasting time collecting and preprocessing the raw data. Researchers without specific knowledge in GIS or remote sensing can also benefit from the EVE system, given that the output data can be interpreted with common statistical tools. The standardization of the processes involving data acquisition and production, on a well-defined spatial and temporal scale, reduces possible errors in the data pre-processing and provides a general harmonization of the data. At present, the EVE system is stable and up-to-date; however, the EVE system structure and its intrinsic modularity permit potential future extensions and improvements, such as integration with new datasets and processing (e.g., new environmental indexes), without having to re-engineer the entire system. For instance, the temporal coverage of the GPM has been planned to be extended to the 2010–2014 period, permitting: (i) the reduction of missing data, especially at the Country level, (ii) the enhancement of the rasters spatial resolution (from 27 to 10 km), and (iii) the increase of the dataset consistency. With reference to VBDs, other important variables include the air humidity (1, 67), even though there are only few providers or project that offer this information, and with a sub-optimal spatial and temporal scale (e.g., https://www.primavera-h2020.eu/about/project/). Alternatively, the relative humidity data obtained from meteo stations could be used for deriving a local modeled continuous dataset. Each present of future module, while exploiting common intermediate functions and outputs, is dedicated to the production of specific outputs at multiple scales, optimizing the computational requirement.

A potential improvement would consist in storing the whole EVE datasets inside a dedicated Database, in order to better manage their acquisition and subsequent analysis. Given the rapid environmental changes and the increase in the quantity of available data, innovative solutions are needed to elaborate and produce accurate outputs for the epidemiologists and decision makers. This would prompt toward a data cube approach (68), which could improve the efficiency for both data extraction and analysis, once combined with Machine Learning techniques and algorithms.

Data from the Copernicus Marine Service portal (CMEMS) have been already included in specific workflows on shellfish farming. More specifically, the sea surface temperature and the oxygen, salinity, chlorophyll and phytoplankton concentrations products helped in the monitoring activities in a mussel farm located in the Northern Adriatic Sea. Automating the acquisition of such datasets could be helpful to implement an early-warning system.

The platform presented in this work represents a valid and convenient alternative method for obtaining, pre-processing, and extracting environmental and climatic data needed for studies oriented to the analysis of eco-epidemiological aspects of infectious diseases. Although the needs of the typical end-users are, in most cases, fulfilled by the acquisition of pre-processed data available on the various aforementioned official providers, and the availability of online services allows analyzing data directly on the web through quite common frameworks, the currently available tools may be not sufficient to meet all the specific needs of researchers and analysts. For example, analyses focussed on a local scale would require data which are not available on such platforms, or the services offered by them might have some limitations that are too restrictive for the collection of the necessary data, so proper and effective alternatives have to be evaluated. Furthermore, if the need of data collection is frequent as well as the availability of ready-to-use datasets in the required format, without the concern related to technical aspects of data acquisition and harmonization, the development of an in-house system such as the one presented here could represent a valuable and advantageous solution. From these considerations, it follows that a technical team with a multidisciplinary background (IT, GIS, basics of statistics) is mandatorily required for the maintenance of the implemented system, which in turn might represent a limit in terms of human and economic resources available. However, the investment in a dedicated support team represents a worthwhile opportunity to keep the technologies constantly up-to-date, allowing the epidemiologists, researchers and analysts to entirely focus on their study and research goals.

## Data availability statement

The original contributions presented in the study are included in the article/supplementary material, further inquiries can be directed to the corresponding author.

## Author contributions

MM, PM, and NF generated the original idea and system concept. MM, GMar, and MB implemented the system and processed the data. PM, DF, FS, and GMan provided support for the veterinarian and sanitary aspects. CC, DF, and FS tested the system. All author participated to the drafting and revision of the manuscript. All authors contributed to the article and approved the submitted version.
